# *Fusarium oxysporum* and *Colletotrichum musae* Associated with Wilt Disease of *Coffea arabica* in Coffee Gardens in Saudi Arabia

**DOI:** 10.1155/2022/3050495

**Published:** 2022-09-12

**Authors:** Zarraq AL-Faifi, Wail Alsolami, Emad Abada, Habib Khemira, Ghaliah Almalki, Yosra Modafer

**Affiliations:** ^1^Centre for Environmental Research and Studies, Jazan University, Jazan 45142, P.O.Box: 114, Saudi Arabia; ^2^Department of Biology, Faculty of Science, Jazan University, Jazan 45142, P.O.Box: 114, Saudi Arabia

## Abstract

This study aimed to identify if *Fusarium* and *Colletotrichum* species are linked to coffee leaf wilt symptoms (*Coffea arabica* L.) gardens in Jazan region, Kingdom Saudi Arabia. The symptomatic wilted leaves and shoots were collected from Jazan Mountain Region Development Authority (JMRDA) farm in jabal Fifa. Samples of roots and leaves tissues were plated on Dox' Agar medium and incubated for one week at 24^o^C. Two morphologically different fungus colonies grew on the medium. A PCR-based method was used for the molecular amplification and characterization of the fungi using a 18SrRNA specific primer. 1323 and 1501 bp PCR products were obtained by using the 1% agarose gel electrophoresis. The sequence analysis and genbank homology revealed that the present fungi were *Fusarium oxysporum* and *Colletotrichum musae* with 99 and 98% similarity, respectively. Both fungi sequences were submitted to the genebank under accession numbers OP010081 and OP010082, respectively. This is the first report of these two genera of fungi infecting the roots and leaves of coffee trees in Jazan Region of Saudi Arabia and suggests that other fungus species may play a significant role as diseases in other coffee-producing areas.

## 1. Introduction

Soil-born *Fusarium* poses a particular challenge to growers handicapped by the limited areas suitable for coffee growing and the susceptibility of available cultivars.

For at least five hundred years, Saudi Arabia's southern region (provinces of Al Baha, Aseer, and Jazan) has been regarded as a conventional setting place for coffee farming. According to JMRDA's statistics, around 100,000 trees are present in the district of Addayer, Edabi, AlRayth, and Faifa, accounting for about 84% of the total average in KSA. The entire production was about 500 T of green coffee beans in 2021. Almost all Yemeni and Saudi Arabian coffee comes from a prehistoric “heirloom” coffee arabica type originally naturalized centuries ago. This is the only known coffee converging across Saudi Arabia's southwest regions [1]. The main cultivars are Kholani, Shadawi, and Udaini [2]. The coffee gardens are located on mountain terraces at altitudes ranging from 1200 to 1700 m above sea level. Because of the limited area of the gardens, the growers tend to plant the trees 1-2 m apart, resulting in dense thickets when older; thus, inter-shading is often a problem resulting in hard to control pockets of pests and diseases. Recent plantings adhere better to modern planting rules and commonly have a 2 × 3 m spacing [3]. Most gardens are managed organically, without synthetic fertilizers or pesticides; growers frequently use goat manure as the sole soil amendment and keep their terraces free of weeds by fragment filling [4].

Whether the inoculation on damaged or complete roots, López-Lima et al. [5] discovered that *Fusarium oxysporum* isolates from coffee corky roots could colonize the xylem of roots of coffee seedlings. The *Fusarium* genus includes both non-pathogenic and pathogenic strains found in the soil. Many economically significant crops are affected by the latter, which causes plant wilt and root infections [6]. There are more than 120 *formae speciales* (ff. spp.) that have been identified so far, all pathogenic to only one or a few host plant species [7, 8]. Most *Coffea arabica* cultivars are susceptible to *Fusarium* spp., which causes wilt of trees and dry root rot.

Moreover, in some parts of East Africa, wilting and death of coffee trees result from root infection by *Fusarium solani*. In Yemen, this species was reported on phyllosphere and phylloplane of qat, banana Dwarf Cavendish, and potatoes var. Desiree. Many *Colletotrichum* species have been identified as harmful to coffee trees in Mexico. A similar diversity of pathogenic organisms has been found on coffee plants in Asia by Nguyen et al. and Damm et al. [9, 10] (*C. gloeosporioides*, *C. karstii*, and *C. siamense*). In South America, three *Colletotrichum* spp. are connected with coffee plants: *C. gigasporum* and *C. siamense* in Colombia [11, 12] and *C. gloeosporioides* and *C. siamense* in Brazil. The first report of coffee anthracnose in Mexico was made in Puebla, a warm, humid climate, in 1952 (CNC, 1952). The pathogen suspected of causing it was *C. gloeosporioides* [13].

The area is similar to Jebel Faifa in Jazan. This research aimed to identify the pathogen responsible for the extensive withering of coffee trees in Jazan Region of Saudi Arabia in late summer 2021 and the previous year.

## 2. Materials and Methods

### 2.1. Sample Collection

Roots and chlorotic leaves were collected from coffee plants showing severe wilt symptoms growing in the JMRDA's farm in Faifa Mountains in Jazan Region. Roots and leaves were collected from wilted trees from three sites in Jazan Region: JMRDA's farm in Jebel Faifa (17˚15′20″N, 43˚06′21″E; elevation 1541m a.s.l.) in Faifa district, a garden in Habes (17˚19′00.6″N 43˚11′30.1″E; elevation 1484m a.s.l.) in Eddair district, and a third garden from Jebel Smad (17˚20′48″N, 43˚01′50″E; elevation 1450m a.s.l.) in Eddair district. The monthly mean minimum and maximum temperatures were 14°C and 33°C, respectively. The orchard's soil is sandy loam with a slightly alkaline pH (7.7). The orchard floor was maintained free of weeds by periodical disking and manual weeding. At this location, coffee trees flower in early spring and the cherries ripen in November and December. Root and leaf samples were collected from 2-3 trees at each site. The samples from all these sites were bulked to make one composite sample of roots and one sample of leaves. These samples were sanitized by immersing them in a 5% Clorox solution for 1-2 minutes and then rinsing them with sterile distilled water. The leaves were sliced into 1 cm^2^ pieces, while the roots were cut into 1 cm long pieces. Sterilized samples were placed in 12 cm Petri dishes on top of Dox medium (Emmons, 1980). Each tissue had three replicate plates. The plates were incubated at 25°C for seven days to see fungi grow. The morphological studies were performed after seven days of incubation at 25°C. A light microscope examined the colony form, color, strength, spore shape, color, and size [14].

### 2.2. Molecular Characterization of Fungal Isolates

#### 2.2.1. DNA Extraction

60 mg of mycelium was put in 1.5 mL Eppendorf tubes containing sterile glass microspheres. Then, 500 *µ*L extraction buffer and 2.5 *µ*L RNAse A were added (10 mg mL^−1^). The tubes were vortexed vigorously before incubating at 45°C for 40 minutes. After that, 150 *µ*L of 5M potassium acetate was added, and the mixture was incubated for 15 minutes on ice. After centrifuging the tubes at 11,000 rpm for 5 minutes, the supernatant was transferred to a clean Eppendorf tube. The DNA was precipitated by adding cold isopropanol, incubating for at least two hours at −20°C, and centrifuging for 5 minutes at 11,000 rpm. The supernatants were discarded, and the DNA pellets were centrifuged for 5 min in 500 *µ*L cold ethanol. In the end, 50 *µ*L of Mili *Q* water was added to the DNA pellets [15].

#### 2.2.2. PCR Amplification

PCR primers capable of amplifying the 18SrRNA gene of all isolated fungus were chosen to amplify the 18S rRNA region: 1A (5′-AACCTGGTTGATCCTGCCAGT-3′) and 564R (3′-GGCACCAGACTTGCCCTC-5′). PCR conditions were an initial denaturation at 95°C for 5 min, followed by 30 cycles at 95°C for 55 s, 54°C for 55 s, and 72°C for 60 s. The PCR product's size was estimated using 1 *µ*L of the product which was electrophoresed on a 1% agarose gel. Ethidium bromide was used to stain the gel and then photographed using a UV transilluminator. The PCR products were sequenced by the Macrogen CIA, South Korea (https://dna.macrogen) [16].

#### 2.2.3. Phylogenetic Analysis

*Colletotrichum* and *Fusarium* isolates were phylogenetically studied with the NCBI BLAST (https://blast.ncbi.nlm.nih.gov) by comparing the 18SrRNA sequences against the NCBI database. MEGA version 7.0.26 software was used to modify the DNA sequence, align it with ClustalW, and concatenate it. The maximum likelihood method was used to create the phylogenetic tree.

## 3. Results

### 3.1. Sample Collection and Isolation

**Figure 1:**
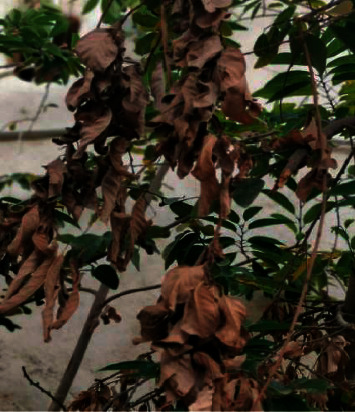
Wilt symptoms on leaves of coffee tree in Jazan Region.

The local arabica coffee varieties in southwestern Saudi Arabia appear susceptible to *Fusarium* wilt. Some growers lost about a quarter of their trees in fall 2021. The yellowing and desiccation of the leaves start from the outer shoot tips and progress inward. The cherries shriveled and failed to mature. The cherries shriveled and failed to mature that lead the death of the entire plant. In this study, samples of the leaves and roots were collected from coffee trees that showed symptoms of pathological wilt (Figure 1).

**Figure 2:**
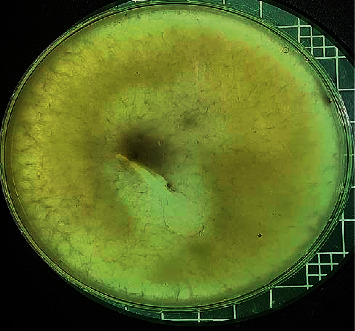
Colonies of *Fusarium oxysporum* from coffee leaves after 7 days of incubation on Dox's medium.

**Figure 3:**
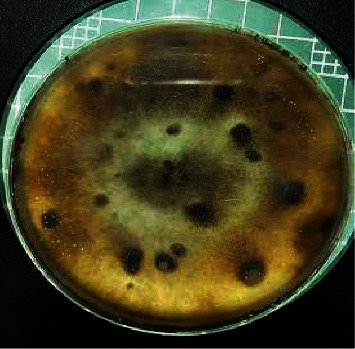
Colonies of *Colletotrichum musae* from coffee leaves after 7 days of incubation on Dox's medium.

When segments of wilting leaves and roots were cultured on Dox's medium designated for the isolation of fungi and left to grow for seven days, two types of fungal colonies appeared around the roots, given the symbol *R* (Figure 2), and a fungal colony around the leaves was given the symbol *L* (Figure 3).

### 3.2. Morphological Study

**Figure 4:**
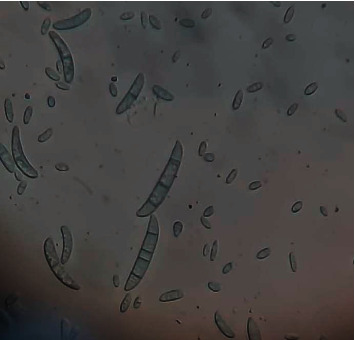
The macroconidia of *Fusarium oxysporum.*

According to the microscopic study, the fungal colony R was characterized by a pale, dark to white colony color with a slightly curved and thick macroconidium. The apical cell morphology was tapered with a slight hook, while the basal morphology was foot-shaped. The mean length of macroconidia was 34 *µ*m, and the mean width of macroconidia was 4 *µ*m; finally, the macroconidia septation was 3 (Figure 4).

**Figure 5:**
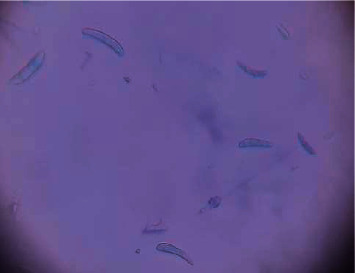
The macroconidia of *Colletotrichum musae.*

Colony L was loose with white aerial mycelia that turned light orange. Many acervulus-like black masses are connected. The conidia were hyaline, aseptate, mostly ellipsoid, and 14 × 7 *µ*m in size (Figure 5).

### 3.3. Molecular Characterization and Homology Study

**Figure 6:**
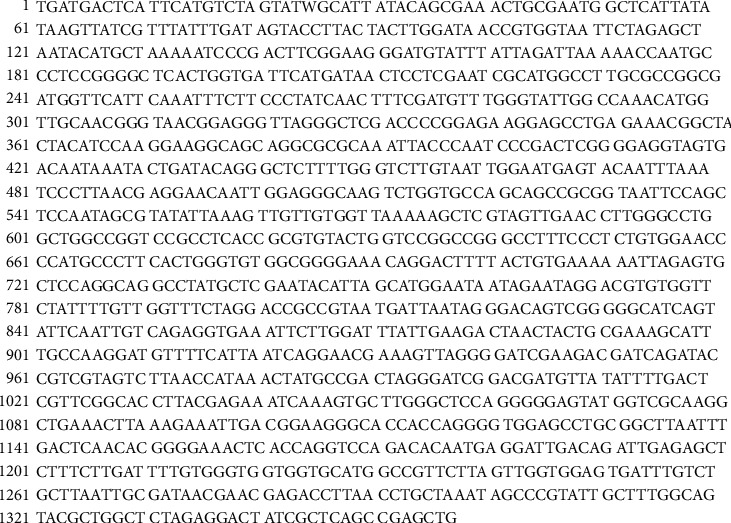
The 1321 bp 18SrRNA partial sequence of *Fusarium oxysporum*.

**Figure 7:**
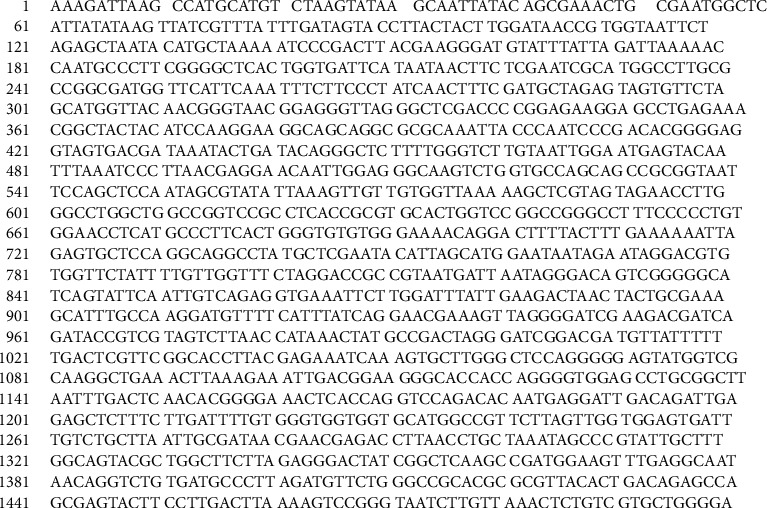
The 1500 bp 18SrRNA partial sequence of *Colletotrichum musae.*

**Figure 8:**
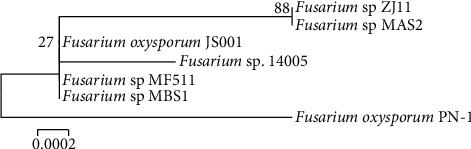
Phylogenetic tree of *Fusarium oxysporum* isolates associated with coffee wilt symptoms.

**Figure 9:**
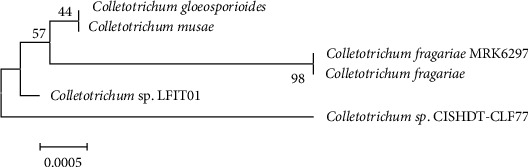
Phylogenetic tree of *Colletotrichum musae* isolates associated with coffee wilt symptoms.

Based on the PCR analysis and agarose gel electrophoresis, the PCR products of samples R and L were 1321 bp (Figure 6) and 1501 bp (Figure 7), respectively. The sequence gene homology study of R and L samples using blast genbank (https://blast.ncbi.nlm.nih.gov) indicated that the R sample is related to genus *Fusarium oxysporum* with 99% homology (Figure 8), while the *L* sample was related to *Colletotrichum musae* with 98% homology (Figure 9). Both fungi sequences were submitted to the genbank under accession numbers OP010081 and OP010082, respectively (https://www.ncbi.nlm.nih.gov/nuccore/OP010081,OP010082).

## 4. Discussion

The present study identified the microorganisms which appear to cause the wilt disease in the *Coffea arabica* trees in Jazan Region. In recent years, many farmers have complained about the wilt and death of their coffee trees without knowing the cause. Interestingly, the gene bank homology study indicated mixed infections in the roots and leaves of the coffee tissue samples with *Fusarium oxysporum* and *Colletotrichum musae*, respectively; this is quite common. The root-knot nematode (RKN) *Meloidogyne* spp. and the fungus *Fusarium* can cause disease complexes caused by the combination of these two things. This can cause damage to a lot of important crops around the world. For example, coffee can be damaged by the variety of *Meloidogyne* spp. and *Fusarium* spp. [17]. Also, in the International Mycological Institute's herbarium, 20 species of *Fusarium* have been identified on coffee specimens [18]. *Fusarium oxysporum*, *Fusarium solani*, *Fusarium stilboides,* and *Fusarium xylarioides* are economically important pathogenic species. *F. xylarioides* causes coffee tracheomycosis, a fungal disease that destroys the plant's vascular system, causing it to wilt and finally die [19]. Mishra et al. determined the presence of a population of *Fusarium* spp. in the soil of guava plantations and linked it to the occurrence of wilt. Colletotrichum infections in coffee are particularly harmful since they directly impact yield [15]. In several countries, *Colletotrichum* species have been reported to damage coffee in many ways, such as infections (causing anthracnose symptoms), endophytes, and epiphytes. So far, 16 *Colletotrichum* species have been identified as threatening coffee crops worldwide. *C. karstii*, *C. acutatum*, *C. capsici*, *C. boninense*, *C. walleri*, and *C. gloeosporioides* were discovered in Vietnam; three were discovered in Thailand: *C. capsici*, *C. acutatum*, and *C. boninense*, two in Angola: *C. fragariae* and *C. cuscutae*; and one species in each of the following five countries: Australia (*C. theobromicola*), Colombia (*C. gigasporum*), Costa Rica (*C. costarricense*), Fiji (*C. theobromicola*), Australia (*C. queenslandicum*), and Kenya (*C. kahawae* subsp. kahawae) [9–11, 21, 22]. Of these 16 species, *C. kahawae* subsp. kahawae Waller and Bridge, which is limited to Africa, is considered a significant concern for coffee growth. It has been reported in Malawi, Kenya, Angola, Tanzania, and Cameroon [22]. It causes coffee berry disease (CBD), an extremely destructive disease that causes premature fruit mummification and fruit drop [23]. *Colletotrichum* spp. diseases cause signs of anthracnose on fruits, stems, and leaves; they have been discovered using morphological and molecular methods [10, 24, 25]. It is particularly problematic that their CBD is now present in Saudi coffee gardens because the main local cultivars appear susceptible. Presently, there are no breeding programs to introduce CBD resistance into these cultivars. It was formerly believed that only *C. coffeanum*, which was subsequently determined to be *C. gloeosporioides* [13], could cause infection of coffee trees. *C. gloeosporioides* has been reported to affect coffee crops in Vietnam and Brazil [26]. Also, *C. gloeosporioides* possibly infect a wide range of other hosts in various countries [27].

We reported here for the first time the presence of *Fusarium oxysporum* and *Colletotrichum musae,* which appear to be responsible for the wilt of *Coffea arabica* trees in Jazan Region. The immediate plans shall include controlling both fungi using an approach to limiting these diseases' spread.

## Data Availability

The data that support the findings of this study are available from the corresponding author upon reasonable request.
